# Case Report: Comparison of Plasma Metagenomics to Bacterial PCR in a Case of Prosthetic Valve Endocarditis

**DOI:** 10.3389/fped.2020.575674

**Published:** 2021-01-07

**Authors:** Joshua A. Lieberman, Caitlin Naureckas Li, Gabriella S. Lamb, David A. Kane, Mary K. Stewart, Ruslan A. Mamedov, Brad T. Cookson, Stephen J. Salipante

**Affiliations:** ^1^Department of Laboratory Medicine & Pathology, University of Washington, Seattle, WA, United States; ^2^Division of Infectious Diseases, Department of Pediatrics, Boston Children's Hospital, Boston, MA, United States; ^3^Department of Cardiology, Boston Children's Hospital, Boston, MA, United States; ^4^Department of Microbiology, University of Washington, Seattle, WA, United States

**Keywords:** metagenomics, endocarditis (all infectious agents), 22q11 deletion syndrome, broad-range 16S rDNA PCR, cell free DNA (cfDNA), next-generating sequencing

## Abstract

Molecular assays for infectious diseases have emerged as important clinical decision-making tools. Unbiased, metagenomic next-generation sequencing is a novel approach holding promise to detect pathogens missed by conventional modalities and to deconvolute admixed nucleic acid sequences from polymicrobial infections in order to identify constituent pathogens. Recent studies have raised concerns about the clinical impact of metagenomics assays and whether their expense is justified. Here, we report a case of polyclonal *Streptococcus cristatus* endocarditis in a 14-year-old woman with a history of Tetralogy of Fallot. Three sets of admission blood cultures and a commercial plasma metagenomics assay were negative for pathogens, despite persistent vegetations observed on the valve during a later procedure. Multiple strains of *Streptococcus cristatus* were identified from the explanted valve by amplicon-based 16S rRNA sequencing, confirming the patient had received appropriate antibiotic therapy. This case highlights limitations in the use and interpretation of clinical metagenomics for infectious disease diagnosis and indicates that the clinical yield of these tools may depend upon infection type and anatomic location.

## Introduction

The recent development of metagenomic next-generation sequencing (mNGS) methods to diagnose infections has created exciting opportunities to unbiasedly detect bacterial, viral, and eukaryotic pathogens from paucicellular patient specimens including cerebral spinal fluid (1–3), blood plasma (4–8), and urine (9). mNGS consists of agnostic DNA, RNA, or total nucleic acid sequencing followed by bioinformatic classification of sequence reads against databases of known organisms. Applications of this approach have ranged from the detection of occult pathogens during fulminant infection (3), chronic disease monitoring in at-risk populations like Cystic Fibrosis patients (8) and diagnosis of infection following antibiotic exposure (10).

Nevertheless, only a few clinical mNGS assays are currently available from commercial or academic providers (3, 11), and their clinical utility remains uncertain (5). Recent studies examining the performance characteristics of mNGS for pathogen detection have estimated clinical sensitivity above 70% (1, 10) and specificity approaching 99% (1, 12). Nevertheless, other large, longitudinal studies of mNGS have demonstrated positivity rates at 32% (6), 49% (13), or 61% (4). The proportion of mNGS results that led to changes in clinical care similarly ranged from as low as <10–14% (4, 13) to as high as 61.4–65% (3, 6, 14) of cases. Clinical investigations have also highlighted the risk of both false negative mNGS testing (6) and over-reporting of clinically unimportant microbes (13), with potentially negative impacts to patient care, including unnecessary treatment (4). Although some of the more pessimistic assessments of mNGS clinical utility have been contested (7), the cost and limitations of the assay merit careful stewardship and selective utilization (4, 7).

The uncertainties surrounding mNGS assay utility leave it unclear if and when clinicians treating infectious diseases should prioritize mNGS testing over conventional molecular diagnostic tools. While a single class of broad-range PCR (e.g., bacterial) or organism-specific PCRs detect fewer pathogens than mNGS, such assays are reported to be at least as sensitive (4), less expensive (15, 16), and can be performed directly on material with a preponderance of human cells, including infected body fluids, tissues, explanted devices, and, in many cases, formalin-fixed paraffin embedded tissue blocks (16, 17).

Here we present a patient who developed prosthetic valve endocarditis due to *Streptococcus cristatus* for whom blood cultures and a commercial mNGS assay to detect microbial cfDNA from plasma was negative for pathogens, while a broad-range bacterial PCR (18) performed on the explanted valve identified the etiologic agent. These discordant results highlight the importance of patient and specimen selection for the optimal application of molecular diagnostic techniques.

## Case Description

**Figure 1:**
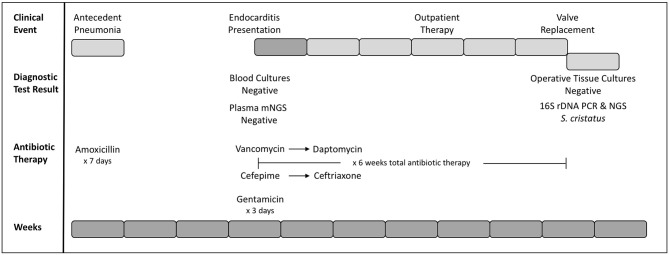
Timeline of clinical and diagnostic events. Weeks since initial presentation (antecedent pneumonia) are presented as gray blocks of 1 week each (bottom row). Clinical events are presented (top row) with inpatient weeks in dark gray and outpatient weeks as light gray blocks. Diagnostic testing and corresponding results, as well as selection and duration of antibiotics are indicated.

The patient was a 14-year-old female with history notable for 22q11 deletion syndrome with Tetralogy of Fallot (TOF), repaired cleft lip and palate, and bilateral hearing loss. Management of her TOF was atypical as she was adopted from another country. She originally underwent placement of a central shunt at age 2-years and complete TOF repair with a bioprosthetic pulmonary valve at age 6-years. A transcatheter pulmonary valve (TPV) was subsequently placed at the age of 12-years. She had a remote history of tooth extractions, but intact dentition at presentation. She was in her usual state of health until approximately 2½ weeks prior to admission (Figure 1). At that time, she developed fever, cough, nasal congestion, and right-sided abdominal pain. A chest x-ray was performed and interpreted as showing a right lower lobe pneumonia. She received a seven day course of amoxicillin, with resolution of her fever.

The patient subsequently presented to her cardiologist's office for routine annual follow-up. An echocardiogram revealed new right ventricular outflow tract obstruction, moderate right ventricular hypertension, and an organized mass associated with the TPV. Given concern for endocarditis, she was admitted to the inpatient cardiology floor for further evaluation (Figure 1). Three sets of large-volume aerobic and anaerobic blood cultures were obtained and the patient subsequently started on a course of empiric vancomycin (4 days, 15 mg/kg q8h IV), cefepime (4 days, 50 mg/kg q8h IV), and synergistic gentamicin (2 days, 1 mg/kg q8h IV). The decision was made to discontinue gentamicin on hospital day three, as the risks to her residual hearing were felt to outweigh the benefits.

Blood cultures drawn at the time of admission prior to IV antibiotic administration as well as those collected on the following 4 days consistently recovered no microbial growth. mNGS testing of patient blood plasma was pursued through a commercial vendor (Karius), and failed to detect any microorganisms at statistically significant levels. No subthreshold microbial DNA was reported. Targeted *Bartonella* PCR from the blood (ARUP Laboratories) was also negative.

During the course of hospitalization, it was hypothesized that the patient's endocarditis resulted from an amoxicillin-sensitive organism that had been suppressed by the patient's recent course of amoxicillin. Cefepime was discontinued in favor of more narrow spectrum ceftriaxone, and vancomycin was transitioned to daptomycin for ease of home administration. Following 6 weeks of therapy, the original conduit with the infected TPV was explanted and a new pulmonary valve placed. Direct surgical inspection of the valve noted vegetations, although microbiologic cultures from intraoperative samples were negative for growth. Tissue from the valve was sent to the University of Washington Molecular Microbiology laboratory for further evaluation.

## Diagnostic Assessment

Broad-range bacterial PCR was performed essentially as previously described (18) and DNA extraction was confirmed by amplification of the human beta globin gene. A single pair of broad-range primers targeting the 16S ribosomal DNA (rDNA) amplified two distinct products of 371 and 411 nucleotides (nt) as visualized by capillary electropheresis. Bidirectional Sanger sequencing was performed, respectively, yielding forward and reverse reads of 326 nt (295 bases with quality value > 20) and 335 (303 bases with quality value > 20). BLAST comparison (19) of the contig formed from these reads against the NCBI 16S nucleotide database indicated 99.15% nucleotide identity over the full length of the contig to a *Streptococcus cristatus* sequence (KF933778) and 98.3% identity to a *S. cristatus* type strain (ATCC 51100, NR_042771). The next-nearest matches were to more genetically dissimilar *S. timonensis* strains, with a maximum percent identity of 97.46% nucleotides (NZ_CABKWP010000001).

**Figure 2:**
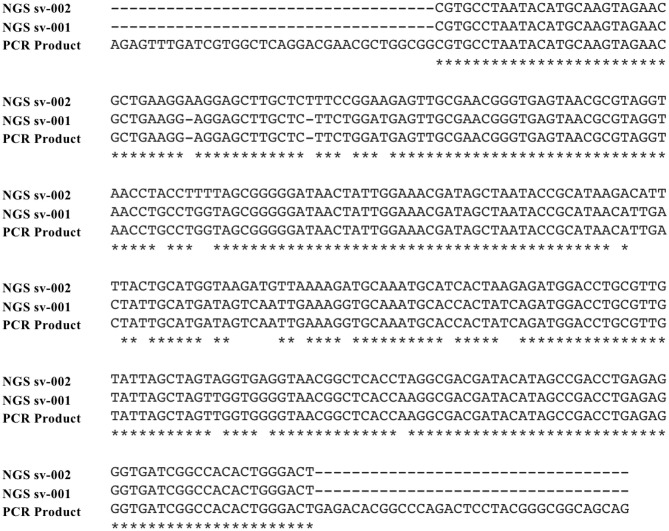
Sequence alignment from Sanger and next generation sequencing reactions. Alignment of the two *S. cristatus* sequences detected by NGS (predominant sequence variant, sv-001; secondary sequence variant, sv-002) and the Sanger sequenced 16S rDNA PCR product are presented with gaps indicated by dashes and nucleotides conserved across all three sequences identified by asterixis.

Given the presence of multiple PCR products, secondary signals in the sequencing reactions, and ambiguity of species identification, clinical NGS of the 16S-amplified product was performed reflexively (20) on an Illumina MiSeq with a 500 cycle kit. The NGS amplicon sequencing assay generated 309,392 paired-end reads, which were reduced to 228,884 after quality filtration and chimera removal (Table 1). The assay identified two sequences (Genbank Accessions MT657947 and MT657948) present in the specimen (97.2% of total filtered reads) but absent from the paired extraction control (NTC, Table 1). These sequences were only 90.88% identical to each other, differing by 2 single nucleotide indels and 26 single nucleotide polymorphisms (Figure 2). The dominant sequence (4.6-fold more abundant, 285 nt) was 100% identical to the corresponding region of the broad-range bacterial PCR product and 98.6% identical to *S. cristatus* type strain ATCC 51100 (NR_042771). The less abundant sequence (287 nt) was 90.88% identical to the original PCR product (Figure 2), but 100% identical to a type strain of *Streptococcus oligofermentans* (AS 1.3089, NR_103943), a heterotypic synonym of *S. cristatus* (21), and to other *S. cristatus* records. These findings supported the diagnosis of a polyclonal endocarditis with two genetically distinct strains of *S. cristatus*.

**Table 1:** Summary of 16S NGS data.

**Classification^a^**	**Sequence variant**	**Total filtered reads**	**Percent of filtered reads**	**Paired NTC^b^ reads**	**Ratio sample: NTC^b^**
**Total reads**	**N/A**	**228,884**	**100**	**12,171**	**18.81**
*S. cristatus*	sv-001	180,991	79.08	15	12,066
	sv-004	1,335	0.58	0	N/A
	sv-006	640	0.28	0	N/A
*S. cristatus* (*S. oligofermentas*-type)	sv-002	39,259	17.15	0	N/A
	sv-009	284	0.12	0	N/A
*Acinetobacter bereziniae* or *A. guillouiae*	sv-005	766	0.33	1,642	0.47
	sv-010	250	0.11	668	0.37
	sv-011	249	0.11	321	0.76
*Cutibacterium acnes*	sv-003	1,904	0.83	4,586	0.42
*Rhizobium radiobacter*	sv-007	448	0.20	449	1.0
Miscellaneous (each sv ≤ 320 reads and ≤0.2% of filtered reads)	sv-008 & sv-012–sv-048	2,758 (aggregate)	1.16 (aggregate)	≤407	*Variable*

a *Classified organisms below the laboratory-established clinical reporting threshold and/or representing reagent contamination are highlighted in gray*.

b *No template control (NTC)*.

## Discussion

The pathogen identified in this case, *S. cristatus*, is a viridans streptococcus and member of the *S. mitis* group (*S. cristatus* clade) (21) that grows in poly-microbial periodontal biofilms, often with other oral streptococci (22–24). Viridans streptococci are an important cause of endocarditis and several cases of endocarditis specifically caused by *S. cristatus* have been reported (25–28). As exemplified by this case, *S. cristatus* endocarditis is frequently sub-acute (25–27), may be poly-streptococcal (25), and should respond to beta-lactam antibiotics (25, 27). Although such infections can occur subsequent to pneumonia (25), cases of *S. cristatus* endocarditis involving native valves without antecedent pathology have also been reported (25, 26). The 16S rDNA NGS sequencing results here highlight inherent challenges in identifying specific *S. mitis* group members owing to significant sequence diversity in the 16S rDNA gene (the target for broad-range bacterial PCR), and multiple name changes that have occurred within this group (21).

This case is notable for the discordance between conventional and mNGS diagnostic techniques. Broad-range bacterial PCR performed on DNA extracted from lesional tissue surrounding the prosthetic valve provided an effective diagnosis. The negative result from plasma mNGS testing was unexpected, given the identification of bacteria from tissue directly interfacing with a high-flow region of the blood stream. Although endocarditis would theoretically provide an optimal situation for release of cfDNA from a pathogen into the blood, *S. cristatus* cfDNA remained below the limit of detection for mNGS. One explanatory hypothesis is that the biofilm matrix of *S. cristatus* trapped extracellular pathogen DNA (22), preventing its release into the blood. Alternatively, pathogen cfDNA may have proven unstable after collection and could have degraded before mNGS testing was performed (15).

It is unclear whether clearance of microbial cfDNA could account for the negative mNGS assay. Little is known about the pharmacodynamics of microbial cfDNA, although reports have detected microbial cfDNA for at least 2 weeks and as long as 36 days after treatment of endocarditis (29, 30). Inferring clearance in this patient's case is complicated because when she was treated for pneumonia, she did not have evidence of endocarditis; rather, she later presented with increased pulmonary valve obstruction and a new vegetation subsequent to treatment, suggesting progression of the infection despite initial therapy with unknown effects on microbial cfDNA concentration. Detection of 16S amplicons from the explanted specimen demonstrates that intact microbial DNA is present, at least within bacteria on the valve.

Regardless of the root cause, this finding is consistent with a recent analysis that reported plasma mNGS was negative in seven cases of endocarditis (4 culture-negative) and provided discordant results in one case (13). These discrepancies illustrate the limitations of microbial diagnosis performed outside the context of infected tissue. Other reported pitfalls of mNGS testing include reporting clinically irrelevant organisms in as many as 45% of positive plasma mNGS assays (13) and potential negative impacts, such as unnecessary treatment or emergency department evaluation (4). These findings raise important questions about the clinical utility of plasma mNGS suggested by earlier studies (31).

All clinically available mNGS assays for pathogens available at present are performed on paucicellular specimens (5, 32). While we are aware of reports of mNGS performed directly on cellular patient specimens [summarized in (32)], we do not anticipate such specimen types will be available for clinical care any time soon. The reported read counts for pathogens in such specimens, when detected, are exceptional low compared to the human nucleic acid—generally much <0.01% of total reads, even when relatively high copy viruses are identified (32)—emphasizing the risk that pathogens will be masked by high-abundance human nucleic acids, the importance of a paired NTC for analysis, and the need for firmly established reporting thresholds.

Laboratory stewardship requires correctly matching patient populations, specimen types, and testing modalities that are most likely to establish a diagnosis while minimizing turnaround time. Such stewardship is particularly important in selecting molecular testing for infectious diseases, both to speed results for acutely ill patients and to contain health care costs as hospitals come under increasing fiscal pressure. While plasma-based mNGS presents opportunities for molecular diagnosis of disease while minimizing invasive procedures (11), in this case it failed to establish the proper diagnosis, despite gross evidence of an infectious process that directly contacted the patient bloodstream. Physicians should be mindful that negative results from mNGS do not rule out the presence of infection, and that in many cases conventional, tissue-based molecular approaches may provide a definitive diagnosis.

## Data Availability Statement

The datasets presented in this study can be found in online repositories. The names of the repository/repositories and accession number(s) can be found below: https://www.ncbi.nlm.nih.gov/genbank/, MT657947 https://www.ncbi.nlm.nih.gov/genbank/, MT657948.

## Ethics Statement

Ethical review and approval was not required for the study on human participants in accordance with the local legislation and institutional requirements. Written informed consent to participate in this study was provided by the participants' legal guardian/next of kin. Written informed consent was obtained from the parent of the patient for publication of this case report.

## Author Contributions

JL, SS, and RM conceptualized the project, prepared the manuscript, and analyzed the data. CN, GL, and DK were responsible for the patient's clinical care and prepared the manuscript. MS and BC prepared the manuscript and analyzed the data. All authors contributed to the article and approved the submitted version.

## Conflict of Interest

The authors declare that the research was conducted in the absence of any commercial or financial relationships that could be construed as a potential conflict of interest.
